# Metabolomic Profiling of Human Spermatozoa in Idiopathic Asthenozoospermia Patients Using Gas Chromatography-Mass Spectrometry 

**DOI:** 10.1155/2018/8327506

**Published:** 2018-02-28

**Authors:** Kai Zhao, Jianzhong Zhang, Zhen Xu, Yue Xu, Aiming Xu, Wei Chen, Chenkui Miao, Shouyong Liu, Zengjun Wang, Ruipeng Jia

**Affiliations:** ^1^Department of Urology, Nanjing First Hospital, Nanjing Medical University, Nanjing, China; ^2^State Key Laboratory of Reproductive Medicine and Department of Urology, The First Affiliated Hospital of Nanjing Medical University, Nanjing, China; ^3^Department of Urology, Peking Union Medical College Hospital, Peking Union Medical College, Chinese Academy of Medical Sciences, Beijing, China; ^4^Department of Urology, Taizhou People's Hospital, Taizhou, Jiangsu, China; ^5^Department of Microbiology, Nanjing Medical University, Nanjing, China

## Abstract

The purpose of this study was to describe the first metabolic profile of human sperm cells through the application of an untargeted platform based on gas chromatography-mass spectrometry (GC-MS). Sperm cell samples from patients diagnosed with idiopathic asthenozoospermia (*n* = 30) and healthy subjects (*n* = 30) were analyzed using a nontargeted metabolomics method based on GC-MS spectroscopy. The mass spectrometric data were collected using multivariate and univariate analyses to identify metabolites related to idiopathic asthenozoospermia. By using metabolomic strategies, we identified 33 metabolites, 27 of which were decreased in the idiopathic asthenozoospermia group compared with the normozoospermic group and six were increased in idiopathic asthenozoospermia. With respect to human sperm cells, some of these metabolites are reported here for the first time. Pathways for nucleoside, amino acid and energy metabolism, and the Krebs cycle were disturbed and were associated with idiopathic asthenozoospermia. The metabolic profiling provides an important first step in studying the pathophysiological mechanisms involved in IAS, and the identified metabolites may become potential biomarkers for its diagnosis and treatment.

## 1. Introduction

Approximately 10%–15% of men are affected by infertility during their reproductive years, yet its etiology remains incompletely understood and nearly half of the cases are considered idiopathic or unexplained [1, 2]. Asthenozoospermia refers to a decrease in sperm motility in the fresh ejaculate and is a common cause of male infertility [3, 4]. Many factors lead to the occurrence of asthenozoospermia, including varicocele, sperm dysfunction, partial obstruction of the seminal tract, infection, or genetic factors. Nevertheless, some asthenozoospermia cases can be idiopathic, and the etiologies of idiopathic asthenozoospermia (IAS) cannot yet be identified by medical testing [5]. As the underlying causes of IAS remain unknown, a comprehensive understanding of the disorder is needed. Metabolomics might be one way of addressing this question.

The study of metabolomics is defined as a quantitative description of all endogenous molecular metabolites within fluids and cells, using multiple forms of spectroscopy and other analytical methods [6, 7]. Metabolites are low-molecular-weight components (<1500 Da) and include sugars, organic acids, amino acids, lipids, nucleosides, vitamins, and minerals [2, 8]. The aim of metabolic profiling is to determine the metabolic products associated with physiologic and pathologic states, and abnormal metabolic phenotypes can indicate the pathophysiology and pathogenesis of disease [6]. At present, nuclear magnetic resonance (NMR), mass spectrometry (MS), Fourier transform infrared (FT-IR) spectroscopy, Raman spectroscopy, and other techniques are applied for the study of metabolomics. NMR spectroscopy is widely used in metabolomics, as this method is rapid and nondestructive, and the sample preparation is simple. Nevertheless, NMR exhibits lower sensitivity and poorer dynamic range compared with MS. In addition, NMR requires greater metabolite concentrations in samples compared to MS. Currently, MS-based metabolomics is the most popular metabolomics strategy [8, 9] and considering the above factors, MS is more suitable for the present study.

In recent years, the use of metabolomics to study asthenozoospermia has focused mainly on human seminal plasma [2, 4], whereas metabolomic studies have been performed using human sperm cells [9]. However, to the best of our knowledge, there are no published reports concerning metabolomic studies of human sperm cells from idiopathic asthenozoospermic patients. In the present study, therefore, gas chromatography coupled to mass spectrometry (GC-MS) was used to analyze the metabolome of human sperm cells from normozoospermic subjects and those diagnosed with IAS. The study of sperm metabolism in patients with IAS provides a more systematic analysis of the pathogenesis and pathophysiology of the disease.

## 2. Methods and Materials

### 2.1. Ethical Approval

The Ethics Committee of the Nanjing Medical University approved this study, and it was performed in accordance with national and international guidelines. Written informed consent was obtained from all participants involved in the study.

### 2.2. Sample Collection

Thirty individual samples were obtained from 30 normozoospermic donors at the human sperm bank of the First Affiliated Hospital of Nanjing Medical University (FAHNMU), and 30 IAS samples were obtained from 30 men with diagnosed IAS who visited the Reproductive Medicine Center of FAHNMU for treatment of infertility. Patients in the experimental group were excluded if they exhibited symptoms or factors that might have caused or contributed to asthenozoospermia, including infections, varicocele, physical and chemical factors, immune and endocrine factors, chromosomal factors or an abnormal karyotype, or other systemic diseases. All the patients and healthy donors denied any bad habits that may affect sperm quality. The average age of the men was 25.6 ± 6.88 years in the normozoospermic group (range: 21–32 years) and 27.3 ± 5.33 years in the idiopathic asthenozoospermia group (range: 21–32 years). The participants were instructed to collect specimens into specific containers following 3–5 days of sexual abstinence. Routine semen analyses were performed according to the guidelines of the World Health Organization (WHO, 2010). The semen parameters for IAS men were as follows: PR < 32%; sperm concentration > 15 million spermatozoa/mL; total motility (PR + NP) < 40%; and sperm morphology (normal forms) > 4%. The characteristics of the sperm samples were showed as in Table 1.

### 2.3. Semen Preparation

Ham's F-10 nutrient mixture (Life Technologies, Paisley, UK) supplemented with 0.6% (w/v) bovine serum albumin (BSA) and 26 mM bicarbonate (NaHCO3) was used to wash all the semen samples at room temperature. Afterwards, the samples were treated by density gradient centrifugation at room temperature, with 80% (v/v) and 40% (v/v) Percoll at a speed of 400 g for 30 minutes. All sperm cell specimens were examined using a phase-contrast microscope, and no other impurities were detected. As the number of sperm cells obtained by centrifugation was inadequate, the spermatozoa of three individuals were combined into one sample. As a result, there were ten samples for each of the IAS and normozoospermic groups. Spermatozoa were washed with cold PBS three times, cryopreserved in liquid nitrogen, and stored at −80°C until further metabolite extraction was performed.

### 2.4. Metabolite Extraction and Derivatization for GC-MS

The sperm cell specimens were thawed and centrifuged at 3000 rpm for 10 min at 4°C. The supernatants were then removed and any remaining PBS was absorbed using filter paper. The cells were mixed with 1.2 mL of cold methanol-water-chloroform (4 : 1 : 1, v/v/v) and 20 *μ*L of the internal standard (0.3 mg/mL 2-chlorophenylalanine in methanol) and then vortexed for 30 s. The cells were disrupted for 6 min using an ultrasonic cell disrupter system (Biosafer900-92, Biosafe, China), and the resulting mixtures were transferred to 1.5 mL Eppendorf tubes and centrifuged at 10,000 rpm for 10 min at 4°C. Five hundred microliters of the supernatant was then transferred to a glass-sampling vial for vacuum drying at room temperature. The residue was derivatized using a two-step procedure. First, 80 *μ*L of methoxyamine (15 mg/mL in pyridine) was added to the vial, vortexed for 30 s, and then incubated at 37°C for 90 min. This was followed by the addition of 80 *μ*L of BSTFA (1% TMCS) and 20 *μ*L of n-hexane, and the vial was incubated at 70°C for 60 min.

### 2.5. GC-MS Analyses and Quality Control

Each 2 *μ*L aliquot of the derivatized solution was injected into the Agilent 7890A-5975C Gas Chromatograph-Mass Spectrometer (Agilent, California, USA) in splitless mode. Separation was carried out on a nonpolar DB-5 capillary column (30 m × 250 *μ*m ID; J&W Scientific, Folsom, CA, USA), with high purity helium as the carrier gas at a constant flow rate of 1.0 mL/min. The GC temperature program began at 80°C, and the temperature was increased by increments of 10°C/min in an oven to 180°C. This was followed by incremental increases of 5°C/min to 240°C and 25°C/min to 290°C and then a final 11-minute maintenance at 290°C. The electron impact ion source was maintained at 260°C with a filament bias of −70 V. Full scan mode (*m*/*z* 50–600) was used with an acquisition rate of 20 spectra/s in the MS setting. A QC sample was prepared by mixing aliquots of all of the samples into a pooled sample, and this was then analyzed using the same method as the analytical samples. The QCs were injected at regular intervals (every ten samples) throughout the analytical process to provide a set of data from which repeatability could be assessed.

### 2.6. Multivariate Data Analyses

The acquired MS data from GC-MS were analyzed by ChromaTOF software (v 4.34, LECO, St. Joseph, MI, USA). Briefly, after alignment with a Statistic Compare component, the CSV file was obtained with three-dimensional datasets that included sample information, retention time-*m*/*z*, and peak intensities. The detectable peaks in spermatozoa using the GC-MS comprised 596 samples, and the internal standard was used for data quality control (reproducibility). After internal standards and any known pseudo-positive peaks (such as peaks resulting from noise, column bleed, and the BSTFA derivatization procedure) were removed from the dataset, and the peaks from the identical metabolites were combined, the detectable metabolites from the sperm cell samples in the GC-MS effluent were reduced to 265. The dataset was then normalized using the sum intensity of the peaks in each sample.

The datasets resulting from GC-MS were separately imported into the SIMCA-P+14.0 software package (Umetrics, Umeå, Sweden). PCA and OPLS-DA were then carried out to visualize the metabolic alterations between the experimental groups, after mean centering and unit variance scaling. VIP was used to rank the overall contribution of each variable in the OPLS-DA model, and variables with VIP > 1.0 were considered relevant for group discrimination. In this study, the default seven-round cross-validation was applied with one-seventh of the samples excluded from the mathematical model in each round, in order to prevent overfitting.

### 2.7. Identification of Metabolites

All of the differentially expressed compounds in the IAS group were selected by comparison with the controls using the multivariate statistical method and the Wilcoxon−Mann−Whitney test. Metabolites with both multivariate and univariate statistical significance (VIP > 1.0 and *p* < 0.05) were annotated with the aid of available reference standards in our laboratory and the NIST 11 standard mass spectral and Fiehn databases linked to ChromaTOF software (v 4.34, LECO, St Joseph, MI, USA). A similarity of greater than 70% was considered appropriate for the reference standards.

## 3. Results

### 3.1. GC-MS Analysis of Sperm Cell Samples

**Figure 1:**
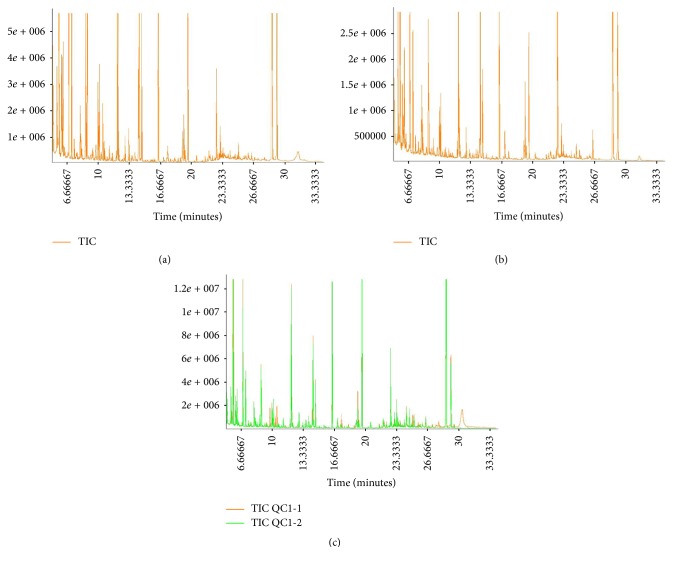
Representative GC-MS total ion chromatograms of the spermatozoa samples from (a) a healthy donor, (b) an idiopathic asthenozoospermic patient, and (c) an overlapped total ion chromatogram of the QC sample.

A typical GS-MS total ion chromatogram (TIC) derived from sperm cell metabolic profiling of the quality control (QC) samples is shown in Figure 1. The retention time and response intensity of the QC sample mass spectrum peak were appropriate, which demonstrated that the analytical method (including the preprocess method and instrumental analysis system) was stable and reliable. Representative GC-MS TIC chromatograms of sperm samples from the IAS group and the healthy control group were also displayed in Figure 1. The majority of the peaks in the chromatograms were identified as endogenous metabolites by NIST mass spectra library, including amino acids, organic acids, and carbohydrates. These metabolites are known to be involved in multiple biochemical processes, especially in energy metabolism [10].

### 3.2. Multivariate Statistical Analysis

**Figure 2:**
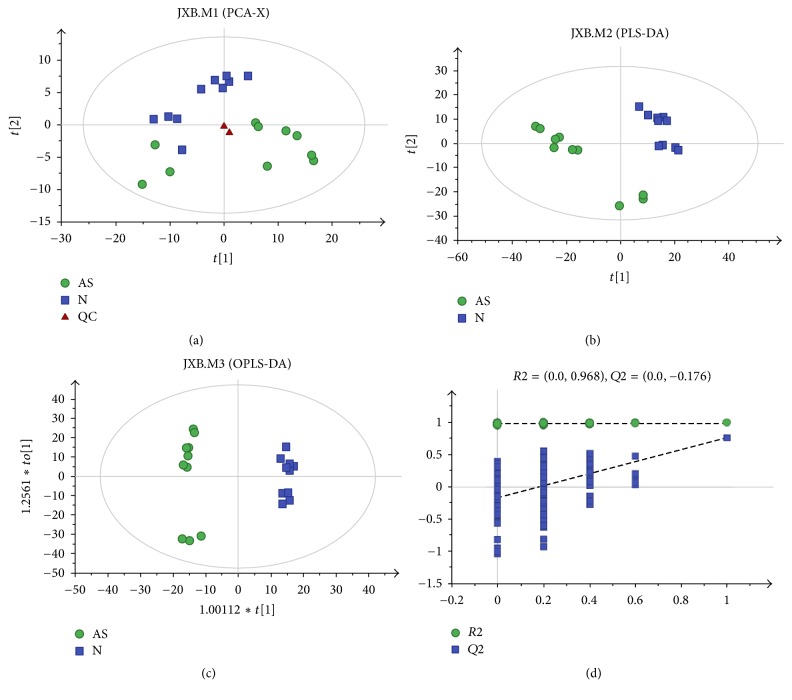
Multivariate statistical analysis for GC-MS based on metabolic profiling of sperm cells from the merged samples of the healthy controls and patients with idiopathic asthenozoospermia. (a) A PCA score plot data from healthy controls (blue) versus idiopathic asthenozoospermia patients (green), (b) a PLS-DA scores plot data from healthy controls (blue) versus idiopathic asthenozoospermia patients (green), (c) a OPLS-DA scores plot data from healthy controls (blue) versus idiopathic asthenozoospermia patients (green), and (d) internal cross-validation plot with a permutation test repeated 200 times.

Principle component analysis (PCA) is a method of unsupervised multivariate statistical analysis, which can determine overall metabolic differences between groups and the variation within the samples in each group. As shown in Figure 2(a), the PCA score plots of all sperm cell profiles from the healthy control and IAS groups demonstrated intrinsic clustering. The clustering between the two groups of samples using PCA indicated that there was a visible difference between the healthy control and IAS groups (*R*^2^*X* = 0.52). In contrast, partial least-squares-discriminant analysis (PLS-DA) is a supervised analysis method.  Figure 2(b) demonstrates that the cumulative R2Y and Q2Y were 0.96 and 0.803, respectively, with the orthogonal PLS-DA (OPLS-DA) model accounting for more clear class discrimination. Moreover, the OPLS-DA model illustrated that the patients with IAS and the healthy controls were separated into two clusters. The OPLS-DA score plot (Figure 2(c)) indicated that the cumulative R2Y and Q2Y were 0.991 and 0.755, respectively. Response permutation testing is a stochastic ranking method that is used to evaluate the accuracy of the model and ensure that the supervised learning method can be obtained without contingency. As demonstrated in Figure 2(d) (200 times; intercept for *R*^2^, 0.0, 0.968; *Q*^2^, 0.0, 0.176), the intercept for the *Q*^2^ regression line was negative. These results indicated that the validation plots could ensure the reliability of the established OPLS-DA models.

### 3.3. Differential Selection of Metabolites

The successful discrimination of the IAS and the healthy control groups led us to search for potential metabolites that might have resulted in the differences between the groups. The multidimensional analysis method, OPLS-DA (combined with the single dimensional analysis method [Student's *t*-test]), was applied to detect differences in metabolites between the IAS and the healthy control groups (variable importance in the projection [VIP] > 1, *p* < 0.05). Using the described methods, 33 metabolites that exhibited significant changes were identified (Table 2).

**Figure 3:**
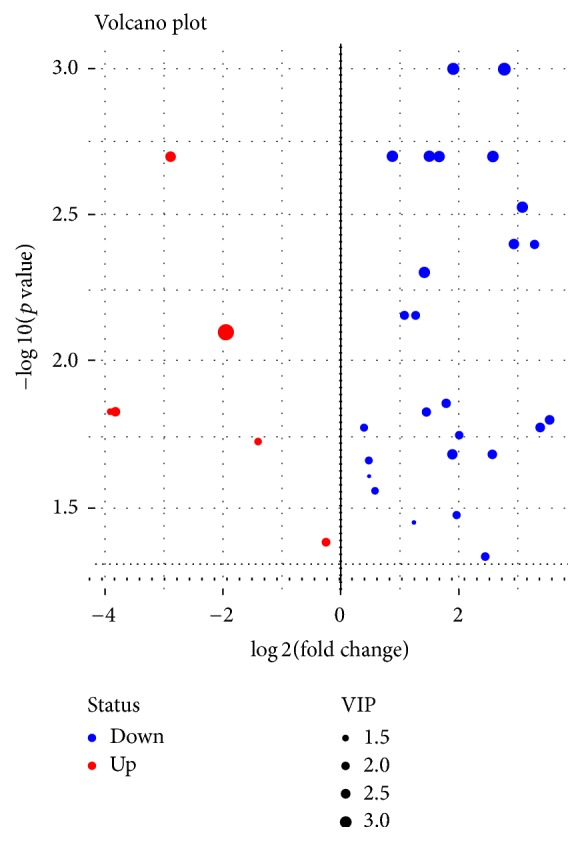
Volcano plot of 33 significantly altered metabolites (*p* < 0.05). *x*-axis: fold change in log⁡2 scale; *y*-axis: −log⁡10 (*p* value); statistical significance was determined by Wilcoxon Signed-Rank test.

Of these metabolites, most were reduced compared with the healthy group, as is illustrated in the volcano plot (Figure 3), including 3-phosphoglycerate, lactic acid, and tryptophan. Only six metabolites were significantly elevated, including zymosterol, dithioerythritol, and orotic acid. Zymosterol is the precursor of cholesterol and is found in the plasma membrane. Orotic acid is synthesized in the body, where it arises as an intermediate in the pathway for the synthesis of pyrimidine nucleotides. Orotic acid is converted to UMP by UMP synthase, a multifunctional protein with both orotate phosphoribosyltransferase and orotidylate decarboxylase activities.

### 3.4. Metabolic Pathway Analysis

**Figure 4:**
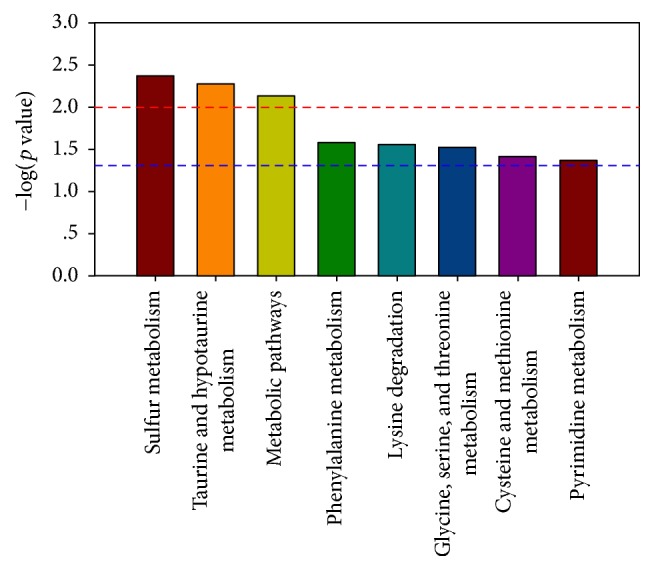
Pathway enrichment analysis of different metabolites. *x*-axis: metabolic pathways; *y*-axis: −log⁡10 (*p* value).

The differentially expressed metabolites were subjected to pathway enrichment analysis to elucidate the mechanisms underlying the metabolic pathway changes in IAS. At present, metabolic pathway analysis is commonly based on the KEGG (http://www.genome.jp/kegg/pathway.html). As shown in Figure 4, the different metabolites were mainly enriched in the sulfur metabolism, metabolic pathways, amino acid metabolism, pyrimidine metabolism, and so forth. The metabolism of some amino acids (such as glycine, serine, and threonine), the glucose/alanine cycle, and gluconeogenesis are important metabolic pathways for the human sperm processes [9].

## 4. Discussion

IAS is considered to be a common cause of human male infertility [11]. It is usually described as a symptom of male infertility because sperm viability and forward motility are reduced [12]. One means of evaluating such infertility parameters is through the use of metabolomics, which can reflect events downstream of gene expression. It is a technique deemed to provide information that is closer to the actual phenotype relative to either proteomic or genomic analyses [13]. With the development of analytical technology and bioinformatics, metabolomics has become a useful tool to study human spermatozoa and, consequently, may constitute a promising source of biomarkers of male infertility. To the best of our knowledge, this is the first time that untargeted metabolomics techniques have been used in idiopathic asthenozoospermia and normozoospermia in order to identify biomedical differences in human sperm cells. The present study is an exploratory study and aims at detecting whether there were any metabolite differences between the idiopathic asthenozoospermic and normozoospermic groups. This was indeed the case. The changes detected mainly included down- or upregulation of nucleoside metabolism, amino acid metabolism, energy metabolism, and the TCA cycle.

Changes in relative levels of several amino acids were found in the idiopathic asthenospermia group compared with the healthy group. The levels of tryptophan, glutamic acid, leucine, and cysteine were significantly decreased in patients with idiopathic asthenozoospermia compared with the normozoospermic group. Tryptophan produces one-carbon units in the process of metabolism, and the primary physiologic function of one-carbon units is to serve as raw material for the synthesis of purines and thymidine and for homocysteine remethylation [14–16]. Thus, the downregulation of tryptophan may have effects on the formation of nucleotides. The levels of glutamic acid and cysteine in sperm cells of patients with idiopathic asthenozoospermia were also significantly decreased. Glutamate is a key compound in cellular metabolism and plays an important role in the body's disposal of excess or waste nitrogen through its deamination. In addition, glutamate reacts with ammonia to produce glutamine, which is catalyzed by glutamine synthetase. Glutamate can give rise to alpha-ketoglutarate, a 5-C Krebs cycle intermediate. Glutamine is an important precursor for the synthesis of amino sugars, proteins, peptides, pyrimidines, and nucleotides and can also supply carbons for oxidation in some cells [17]. Cysteine is an important source of sulfide in human metabolism and is a precursor for the antioxidant glutathione and for iron-sulfur clusters [18].

Some of the amino acids (tryptophan and leucine) were found decreased in idiopathic asthenozoospermic compared with the normozoospermic group. Some of them are called essential amino acids which should be supplied by the diet [19]. The diminished levels of tryptophan and leucine might illustrate a disorder in their metabolism, for example, an increased degradation. Furthermore, the results suggest that adding an additional amount of essential amino acids may be helpful. Other intriguing findings were observed in the idiopathic asthenospermia group where the levels of guanosine and cytidine in sperm cells were attenuated. Guanosine and cytidine participate in the composition of DNA and RNA and have a vital impact on cellular functions. Cytidine is also involved in lipid and carbohydrate metabolism and serves as a substrate in the pyrimidine salvage pathway [20]. In addition to its role as a pyrimidine component of RNA, cytidine has been found to control neuronal-glial glutamate cycling. On the basis of the results above, we suggest that relevant nucleotide metabolism pathways were disturbed in idiopathic asthenospermia.

The levels of 3-phosphoglycerate and lactic acid were decreased in patients with IAS. 3-phosphoglycerate is involved in both the glycolytic and sugar aerobic oxidation pathways, and lactic acid is the final product of glycolysis. One of the downstream products of 3-phosphoglycerate is pyruvic acid, which can provide acetyl-CoA for the Krebs cycle. Sperm motility mainly relies upon ATP to provide energy [21, 22], and glycolysis and oxidative phosphorylation are considered to be the two main ways to generate ATP energy in sperm cells [23]. In addition, occurring within the mitochondria of mammalian sperm cells, the TCA cycle is an important metabolic pathway for the generation of ATP energy. The reduction in 3-phosphoglycerate, glycolysis, aerobic oxidation, and the TCA cycle in sperm resulted in the production of less ATP energy, which might have affected sperm motility. As a result, glycolysis, the TCA cycle, and energy metabolism were disrupted in IAS.

In summary, to the best of our knowledge, this is the first metabolic analysis of human spermatozoa in patients with IAS, and we have used it to demonstrate significant changes at the metabolomic level in sperm cells from these patients. Our understanding of the pathophysiological mechanisms underlying IAS is more comprehensive, and related metabolites identified in the study may serve as potential targets for the diagnosis and treatment of IAS.

## Figures and Tables

**Table 1:** Characteristics of the sperm samples.

Group	Number	Age	Forward motility (%)	Concentration (×10^6^)
NZ	30	25.6 ± 6.88	69.98 ± 7.42	104.74 ± 47.76
IAS	30	27.3 ± 5.33	14.48 ± 5.23	100.85 ± 35.01

Data were presented as means ± SD. NZ: normozoospermia; IAS: idiopathic asthenospermia.

**Table 2:** Metabolites identified by GC/MS analysis of idiopathic asthenozoospermia and normozoospermic groups (*p* < 0.05).

Number	Metabolites	RT (min)	VIP value	*p* value	Fold change	Variations versus healthy controls
1	3-Phosphoglycerate	12.552	2.356	0.001	6.901	↓
2	Lactic acid	13.986	2.104	0.001	1.904	↓
3	Tryptophan	22.841	2.094	0.002	6.051	↓
4	2-Amino-1-phenylethanol	8.318	2.095	0.002	3.196	↓
5	5-Aminovaleric acid	23.320	2.125	0.002	2.847	↓
6	Dithioerythritol	13.233	1.912	0.002	0.135	↑
7	Glutamic acid	9.391	1.628	0.002	1.522	↓
8	8-Aminocaprylic acid	18.527	2.106	0.002	1.847	↓
9	Phytosphingosine	8.151	2.119	0.003	8.511	↓
10	Guanosine	24.600	1.707	0.004	9.838	↓
11	6-Methylmercaptopurine	11.717	1.824	0.004	7.725	↓
12	Norvaline	11.161	1.935	0.005	2.686	↓
13	Leucine	5.815	1.666	0.007	2.123	↓
14	*cis*-Gondoic acid	22.021	1.558	0.007	2.426	↓
15	Zymosterol	29.154	3.483	0.008	0.260	↑
16	Methyl heptadecanoate	9.303	1.651	0.014	3.480	↓
17	Pipecolinic acid	12.805	1.691	0.015	2.762	↓
18	Orotic acid	11.033	1.643	0.015	0.070	↑
19	2-Deoxyerythritol	10.399	1.391	0.015	0.067	↑
20	Phenylethylamine	14.907	1.626	0.016	11.745	↓
21	Guanidinosuccinic acid	9.656	1.496	0.017	1.325	↓
22	*trans*-4-Hydroxy-L-proline	7.807	1.664	0.017	10.533	↓
23	D-Glyceric acid	6.459	1.571	0.018	4.052	↓
24	Benzoic acid	5.602	1.426	0.019	0.380	↑
25	*alpha*-Tocopherol	28.449	1.700	0.021	6.013	↓
26	N-(3-aminopropyl)-morpholine	10.587	1.794	0.021	3.753	↓
27	Picolinic acid	6.470	1.405	0.022	1.394	↓
28	DL-dihydrosphingosine	20.104	1.206	0.025	1.405	↓
29	2-Aminoethanethiol	12.512	1.454	0.028	1.508	↓
30	Cysteine	9.222	1.404	0.034	3.934	↓
31	Cytidine	25.557	1.215	0.036	2.384	↓
32	Ethanolamine	16.080	1.482	0.042	0.838	↑
33	Monoolein	24.574	1.502	0.047	5.496	↓

## Abbreviations

GC-MS: Gas chromatography-mass spectrometry
NMR: Nuclear magnetic resonance
MS: Mass spectrometry
FT-IR: Fourier transform infrared
TIC: Total ion chromatogram
BSA: Bovine serum albumin.
